# Structural plasticity in mesencephalic dopaminergic neurons produced by drugs of abuse: critical role of BDNF and dopamine

**DOI:** 10.3389/fphar.2014.00259

**Published:** 2014-11-25

**Authors:** Ginetta Collo, Laura Cavalleri, PierFranco Spano

**Affiliations:** Department of Molecular and Translational Medicine, University of BresciaBrescia, Italy

**Keywords:** morphology, cocaine, D3 receptor, dendrites, nicotine, ERK, mTOR

## Abstract

Mesencephalic dopaminergic neurons were suggested to be a critical physiopathology substrate for addiction disorders. Among neuroadaptive processes to addictive drugs, structural plasticity has attracted attention. While structural plasticity occurs at both pre- and post-synaptic levels in the mesolimbic dopaminergic system, the present review focuses only on dopaminergic neurons. Exposures to addictive drugs determine two opposite structural responses, hypothrophic plasticity produced by opioids and cannabinoids (in particular during the early withdrawal phase) and hypertrophic plasticity, mostly driven by psychostimulants and nicotine. *In vitro* and *in vivo* studies identified BDNF and extracellular dopamine as two critical factors in determining structural plasticity, the two molecules sharing similar intracellular pathways involved in cell soma and dendrite growth, the MEK-ERK1/2 and the PI3K-Akt-mTOR, via preferential activation of TrkB and dopamine D3 receptors, respectively. At present information regarding specific structural changes associated to the various stages of the addiction cycle is incomplete. Encouraging neuroimaging data in humans indirectly support the preclinical evidence of hypotrophic and hypertrophic effects, suggesting a possible differential engagement of dopamine neurons in parallel and partially converging circuits controlling motivation, stress, and emotions.

## INTRODUCTION

Structural plasticity in neurons can be defined as a series of measurable changes in the morphologically defined components of the neuron, i.e., numbers, size, and composition of soma, dendrites, axons, and synapses, occurring over time and in response to changes in the cell environment. Structural plasticity can also be seen as one aspect of neuroadaptation, a general process present in neurons of specific neural circuits when responding to repeated physiologic stimuli, pathologic agents, or effective doses of pharmacologic substance, including addictive drugs (Ikemoto and Bonci, 2014). These stimuli act by engaging molecular mechanisms that are critical for cell growth and survival, their impact on the cell morphology being defined by the stimulus intensity and by genetic and epigenetic predisposing factors that constitute the neuroadaptive potential of the cells.

Since 1990s neuroadaptation and plasticity have been recognized to be relevant in addiction disorders characterized by chronic misuse of neuroactive substances (Koob, 1992; Nestler, 1992; Di Chiara, 1995; Everitt et al., 2001). In mammals, pharmacologic agents characterized by their addictive properties, for example psychostimulants (e.g., cocaine, amphetamines), opioids (e.g., heroin and morphine), nicotine, cannabinoids, and alcohol, were found to engage dopaminergic neurons of the mesocorticolimbic contingent located in the ventral tegmental area (VTA) (Koob, 1992; Di Chiara, 1995; Koob and Le Moal, 2005; Chen et al., 2010). These neurons produce dopamine (DA) as principal neurotransmitter, project to cortical and limbic brain structures and are involved in regulation of motivation, reward, motor response selection, mood, and arousal. While a large body of experimental findings supports the role for dopaminergic neurotransmission in mediating the addictive properties of these drugs (Koob, 1992; Di Chiara, 1995; Koob and Le Moal, 2005; Kalivas and O’Brien, 2008; Chen et al., 2010), less research was dedicated to the structural changes occurring during exposure to addictive drugs or following their withdrawal. The initial interest on structural plasticity was focused on glutamatergic and GABAergic neurons of nucleus accumbens and prefrontal cortex, i.e., on neurons located in terminal fields of the mesencephalic dopaminergic system (Robinson and Kolb, 1997, 2004; Russo et al., 2010) rather than on their presynaptic side. In dopaminergic neurons structural plasticity was indirectly inferred on the basis of changes in the expression of “marker” proteins though to be involved in structural changes, such as axonal neurofilaments (Nestler, 1992), a phenomenon only later confirmed using morphological techniques (Sklair-Tavron et al., 1996). In fact, by definition, structural plasticity requires morphologic evidence. Dopaminergic neurons are generally identified by immunocytochemistry or immunofluorescence with selective antibodies that recognize tyrosine hydroxylase (TH) or dopamine transporter (DAT; Köhler and Goldstein, 1984). When applied to the *post-mortem* study in mammalian brains, TH immunocytochemistry allows reliable estimate of soma size and neuron counts in substantia nigra (SN, also identified as A9) and VTA (also identified as A10). Conversely, a proper analysis of the dendrite length and branching is not possible, due to the complex overlapping of the dendritic arborizations of adjacent dopaminergic neurons. Visualization of dendrites and dendritic spines of a single neuron requires different approaches, such as the classical Golgi-Cox staining (Juraska et al., 1977) associated with immunohistochemistry (Spiga et al., 2011) intracellular injection with Lucifer Yellow via micropipettes (Sklair-Tavron et al., 1996) or diolistic gene gun delivery of fluorescent dyes (Shen et al., 2008). Dopaminergic neurons can be studied *in vitro* using primary cell cultures from the ventral mesencephalon of rodent embryos or newborns (Shimoda et al., 1992; Collo et al., 2008). The *in vitro* approach allows the simultaneous evaluation of soma size, dendritic arborization, dendritic spines, neurochemistry, and intracellular molecular signaling due to their sparse distribution in the culture dish and their standardized control conditions (Collo et al., 2008, 2012).

In this article we summarize the evidence of structural plasticity occurring in mesencephalic dopaminergic neurons following exposure to addictive drugs, focusing on soma, and dendritic arborization rather than synapses and addressing the key molecular intracellular signaling involved.

## STRUCTURAL PLASTICITY IN DOPAMINERGIC NEURONS AS CELLULAR NEUROADAPTATION: OPPOSITE EFFECTS OF OPIOIDS AND PSYCHOSTIMULANTS

Structural plasticity includes hyperplastic and hypoplastic phenomena, i.e., the increase or decrease of number and size of morphologically defined components of the neuron. In the brain reward circuit, drugs of addiction produce both hyperplastic and hypoplastic phenomena, the former generally associated to psychostimulants, the latter to the use of opioids (for review see Russo et al., 2009, 2010). Since chronic exposure to both opioids and psychostimulants produces behavioral sensitization, compulsive drug taking, and relapse after extinction, at the time of their initial discovery these changes appeared somewhat contradictory, casting some doubts about the relevance of structural plasticity in addictive behavior. Recent findings regarding the role of withdrawal state (Spiga et al., 2010; Mazei-Robison et al., 2011), region-specific changes of synaptic spines (Russo et al., 2010) and differential regulation of endogenous neurotrophins, in particular brain derived neurotropic factors (BDNF) (Russo et al., 2009; Koo et al., 2012), have been advocated as key factors in disentangling this paradox; some possible explanations will be reviewed later in this article.

Chronic exposures to opioids reduce soma size and dendrites of the dopaminergic neurons located in the VTA of adult rodents without reducing the number of neurons (Sklair-Tavron et al., 1996; Spiga et al., 2003; Russo et al., 2007). Opioid-induced hypotrophic effects on soma were observed following either passive dosing or self-administration of heroin or morphine and persist for several weeks during withdrawal. Functionally, chronic exposure to opioids is known to increase VTA neural firing while reduction is observed during withdrawal (Diana et al., 1995; Koo et al., 2012).

Reduced soma size and neural firing were also observed during withdrawal from cannabinoids in rodents (Diana et al., 1998; Spiga et al., 2010). These effects are partially determined by endogenous opioids since acute morphine attenuates the behavioral cannabinoid withdrawal syndrome in mice (Lichtman et al., 2001). Interestingly, chronic exposure to cannabinoids *per se* does not produce change of soma sizes of VTA dopaminergic neurons. Lack of change in the soma size was also recently showed in rats trained to chronically self-administer cocaine, nicotine, and alcohol when sacrificed in presence of drugs (Mazei-Robison et al., 2011). These data do not rule out the possibility of changes during withdrawal or crash after drug taking “binges,” both conditions associated to a functional hypodopaminergic state (Weiss et al., 1992; Melis et al., 2005; Zhang et al., 2012); so far structural effects were not studied.

Cocaine and amphetamine exposures *in vivo* increase dendrite arborization and spines in VTA (Mueller et al., 2006; Sarti et al., 2007). *In vitro* studies on primary cultures of mesencephalic neurons from mouse embryos corroborate this evidence. Dose-dependent increases of soma size, maximal dendrite length, and number of primary dendrites were observed (Collo et al., 2008, 2012). Interestingly, also nicotine was shown to increase structural plasticity of dopaminergic neurons *in vitro*, effect blocked by mecamylamine and dihydro-β-erythroidine but not methyllycaconitine, suggesting the involvement of α4β2 nicotinic receptor (nAChR; (Collo et al., 2013). These nicotinic hetero-receptors expressed in dopaminergic neurons control DA release and are critical for the reinforcing effects of nicotine *in vivo* (Picciotto et al., 1998). Consistently, dopaminergic neurons from the mesencephalon of α4 nAChR-subunit knock out (KO) mice did not show nicotine-induced plasticity (Collo et al., 2013).

Prenatal exposure to either cocaine or nicotine during the last gestational phase (E17-21) was associated with significant increase of soma size of dopaminergic neurons in newborns and young mice (Collo et al., 2012, 2013). Prenatal exposures to cocaine and amphetamines produce long-term changes in the behavior and neurochemistry of the mesencephalic dopaminergic system of offspring assessed as adults (Crozatier et al., 2003; Lloyd et al., 2013), suggesting a possible association between dopaminergic structural plasticity and liability to develop addiction.

## CRITICAL ROLE OF THE BDNF-TrkB SIGNALING IN DETERMINING STRUCTURAL PLASTICITY OF DOPAMINERGIC NEURONS EXPOSED TO ADDICTIVE DRUGS

Neurotrophic factors that bind to the tropomyosin-related kinase B (TrkB) receptor were shown to be of importance in the development of the central nervous system (CNS) and in shaping neuronal morphology of dopamine neurons and other brain circuits (for a review see Ohira and Hayashi, 2009). In particular, BDNF-TrkB signaling has been extensively studied as critical mediator of the structural changes produced by addictive drugs (Russo et al., 2009; Koo et al., 2012). Mesencephalic dopaminergic neurons significantly express BDNF since prenatal time (Baquet et al., 2005). Still present in adult life, BDNF expression can be transiently increased by psychostimulants in VTA dopaminergic neurons (Graham et al., 2007). These increases consolidate and persist over time during abstinence (Pu et al., 2006) and during extinction of drug self-administration and in craving incubation paradigms (Grimm et al., 2003). Infusion of BDNF in VTA induces long-lasting potentiation of cocaine seeking during abstinence (Lu et al., 2004), while BDNF immunoneutralization attenuates the cocaine addictive behavioral effects (Graham et al., 2007). To our knowledge, direct evidence of structural changes in dopaminergic neurons during withdrawal, abstinence and incubation with psychostimulants is lacking in literature. However, in consideration of the well-known BDNF neurotrophic properties on dendrites and soma size, it is possible to speculate that some structural plasticity could occur. Interestingly, GDNF, another neurotrophic factor, increases in VTA during cocaine withdrawal and mediates incubation of cocaine craving (Lu et al., 2009), further supporting possible structural effects.

Almost opposite effects were observed with opioids: morphine reduces BDNF expression in VTA neurons; low BDNF levels were associated with reduced soma size, and local infusion with BDNF normalizes soma size (Sklair-Tavron et al., 1996; Russo et al., 2009). Recent studies using conditional KO mice and optogenetic technology showed that morphine-induced low levels of BDNF in the VTA are associated to hypersensitization of VTA dopaminergic neurons to morphine, whose administration increases firing and DA release, producing conditioned place preference (Koo et al., 2012). Conversely, acute withdrawal and abstinence are associated with increased BDNF expression and TrkB-mediated plasticity changes that are essentials for negative reinforcing effects of morphine withdrawal (Vargas-Perez et al., 2014). Interestingly, the opioid effects on DA release are indirect, mediated by GABAergic inhibitory neurons under glutamatergic control (Bonci and Williams, 1997; Vargas-Perez et al., 2009; Jalabert et al., 2011), suggesting a role also for these neurotransmitters.

The main intracellular pathways activated by BDNF-TrkB signaling are the MEK-ERK, the PI3K-Akt-mTORC1, the PLCy-DAG-PKC/Ca^2+^, and NFkB pathways, all involved in cell survival and growth (Kumar et al., 2005; Russo et al., 2009). These pathways are not only activated by BDNF but also by G-protein coupled receptors (e.g., Girault et al., 2007). Recent evidence indicates that cocaine and nicotine activate both MEK-ERK and Akt-mTORC1 pathways in primary cultures of dopaminergic neurons (Collo et al., 2012, 2013). Phosphorylation in these two pathways was found critical for structural plasticity since pretreatments with selective inhibitors for ERK, PI3K, and mTORC1 block the increase of soma size and dendritic arborization produced by psychostimolants and nicotine (Collo et al., 2013). Conversely, morphine exposure was associated with reduction in Akt levels and phosphorylation, attenuating mTOR-dependent phosphorylation (Russo et al., 2007; Mazei-Robison et al., 2011). The central role of the PI3K-Akt-mTOR pathway in determining soma size of mesencephalic dopaminergic neurons is exemplified by the phosphatase and tensin homolog (PTEN) KO mice. PTEN is a negative regulator of PI3K whose null mutation leads to a constitutive preferential state of activation of Akt-mTORC1 pathway; the result is a massive increase in soma size of dopaminergic neurons already visible in newborns, that persists in adult mice (Diaz-Ruiz et al., 2009). Other mechanisms affecting dendrite and soma size include the modulation of Ca^2+^ levels and the cAMP production, the latter not operated by BDNF. A large body of evidence indicates that Ca^2+^-dependent AMPA and NMDA glutamate receptors regulate dendrite growth in pyramidal neurons and interneurons (Hamad et al., 2011). In dopaminergic neurons NMDA-dependent axonal growth was described as related to CaMKII phosphorylation (Schmitz et al., 2009), while preliminary *in vitro* data indicate a critical role for AMPA receptors. In GABAergic neurons located in the VTA, chronic activation of the cAMP-PKA-CREB was associated with reduced firing and soma size in dopaminergic neurons during morphine withdrawal (Bonci and Williams, 1997; Koo et al., 2012), suggesting an indirect involvement in structural plasticity.

Interestingly, structural changes of soma size and dendritic arborization of dopaminergic neurons are not specific of addictive drugs. In a recent article a reduction of soma size in the VTA was observed in male rats after single and repeated mating episodes (Pitchers et al., 2014). Naloxone treatment reversed soma size reduction and attenuated the longer-term expression of experience-induced facilitation of sexual behavior without affecting its rewarding properties. In another study, an increase of the number, size, and dendritic spines of mesencephalic dopaminergic neurons was associated to exercise and intense motor behavior in rats exposed to moderate dose of dopaminergic neurotoxins (Real et al., 2013), supporting a role for neurotrophic BDNF-TrkB signaling in behaviorally induced structural plasticity.

## DOPAMINE AS NEUROTROPHIC FACTOR: ROLE OF D3 RECEPTOR SIGNALING IN STRUCTURAL PLASTICITY OF DOPAMINERGIC NEURONS

In addition to its role as a neurotransmitter, DA can act as neurotrophic factor. When released in the extracellular space, DA binds to postsynaptic receptors, producing structural plasticity: for example DA increases TrkB phosphorylation via D1 receptor (Iwakura et al., 2008). DA also binds to presynaptic D3, D2_S_, and D5 receptors located on dopaminergic neurons (Zhang and Sulzer, 2012). Functional studies in mutant mice indicate that D2 and D3 receptors are complementary in regulating phasic and tonic dopamine release from dopaminergic nerve terminals in caudate and nucleus accumbens (Le Foll et al., 2005b; Maina and Mathews, 2010). The intracellular pathways activated by the presynaptic DA receptors and related to structural plasticity are only partially understood, being the majority of studies performed in non-dopaminergic cells. Converging findings indicated a primary role for D3 receptors in dopaminergic structural plasticity via phosphorylation of MEK-ERK1/2 and PI3K-Akt-mTORC1 pathways (Cussac et al., 1999; Beom et al., 2004; Collo et al., 2012, 2013). Conversely, D2s receptors inhibit MEK-ERK1/2 pathway and are negatively coupled with adenylate-cyclase (Van-Ham et al., 2007). PLCy activation and β-arrestin non-canonical pathways were described for the D2L splice variant present on postsynaptic neurons (Del’guidice et al., 2011). Finally, less data are available on D5 receptor, whose role has been related to functional plasticity (Schilström et al., 2006; Argilli et al., 2008).

**FIGURE 1:**
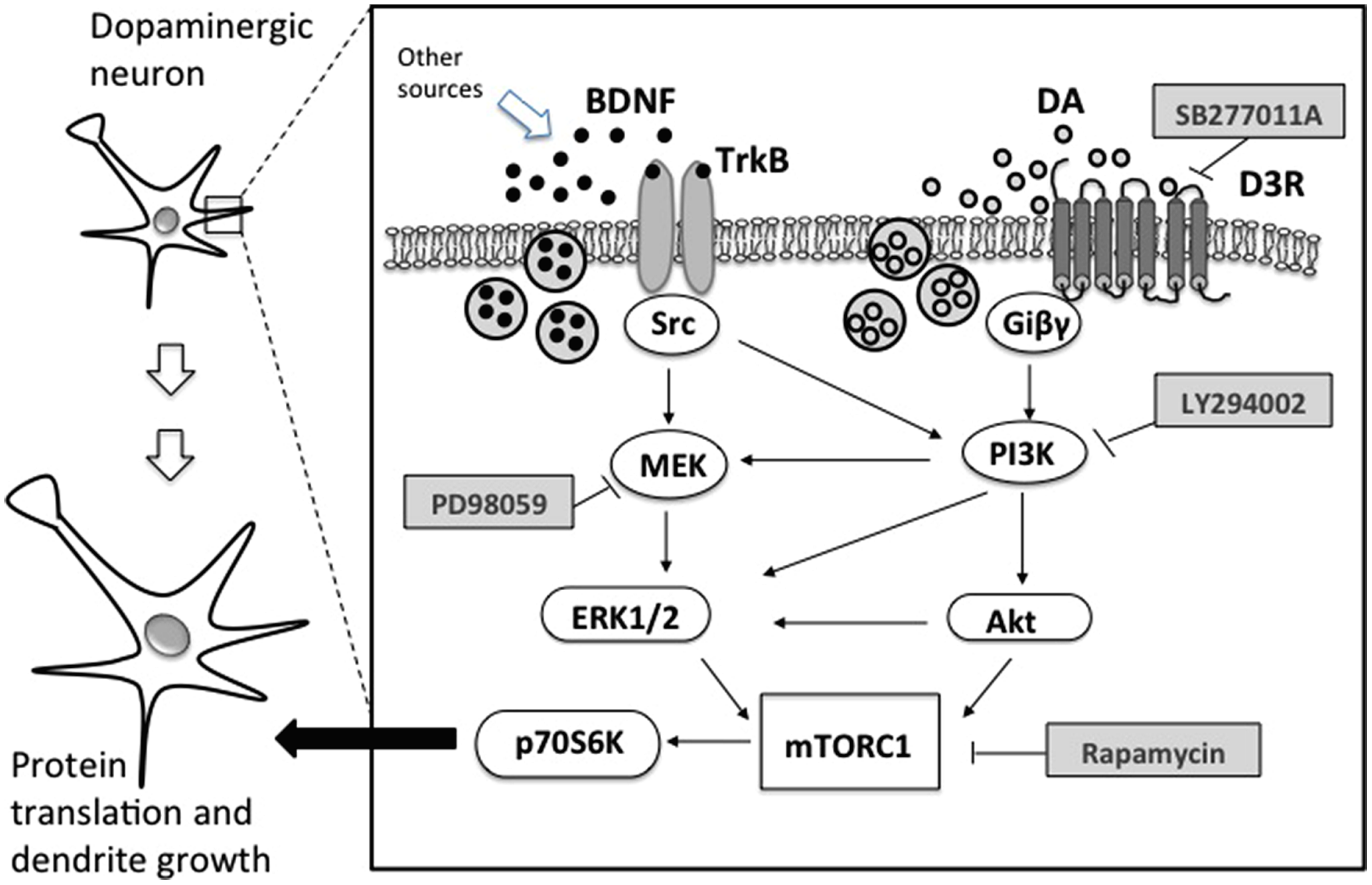
**Schematic representation of relevant intracellular pathways of BDNF and dopamine dependent structural plasticity in dopaminergic neurons.** TrkB, tropomyosin-related kinase B; Src, proto-oncogene tyrosine protein kinase; MEK, mitogen-activated protein kinase; ERK1/2, extracellular signal-regulated kinase; D3R, dopamine D3 receptor; Giβγ, G protein; PI3K, phosphatidylinositol 3-kinase; Akt, serine threonine kinase or protein kinase B; mTORC1, mammalian target of rapamycin complex 1; p70S6K, p70 ribosomal S6 protein kinase; PD98059, MEK inhibitor; LY294002, PI3K inhibitor; rapamycin, mTORC1 inhibitor; SB277011A, selective D3R inhibitor.

Direct evidence linking D3 receptors and structural plasticity was recently obtained in primary cultures of dopaminergic neurons from mouse embryos. Repeated exposure with low doses of D3-preferential agonists, such as quinpirole or 7OH-DPAT, increased soma size and the number and length of primary dendrites (Collo et al., 2008). These effects were also produced by drugs of addiction such as cocaine, amphetamine, nicotine, and ketamine, all known to increase extracellular levels of DA in the VTA. Pretreatments with DA D3 selective antagonist SB277011-A and the non-selective D2/D3 antagonist sulpiride resulted in a blockade of dendrite outgrowth and soma size (Collo et al., 2008, 2012). No structural plasticity was observed when treatments with psychostimulants or nicotine were performed in cell cultures from the mesencephalon of D3 KO mice (Collo et al., 2008, 2012, 2013). When nicotine was repeatedly administered to pregnant D3 KO mice during the last gestational phase, no effect was observed on the soma size of VTA neurons of newborns. Recent evidence suggests that D3 receptors work in concert with BDNF-TrkB signaling. *In vivo* experiment showed that D3 receptor expression depends on the levels of BDNF (Guillin et al., 2003) and that cocaine exposure increases the synthesis of both BDNF and D3 receptors (Le Foll et al., 2005a), while morphine increases the expression of D3 receptors only (Spangler et al., 2003), marking a difference between the two addictive drugs (**Figure 1**).

## HUMAN EVIDENCE OF ADDICTIVE DRUG INDUCED STRUCTURAL PLASTICITY

No direct human evidence of structural plasticity induced by addictive drugs in dopaminergic neurons is currently available. *Post-mortem* studies in cocaine users revealed a 16% reduction of melanized dopaminergic neurons with no reported change of soma size (Little et al., 2009), while a reduction of TH levels in dopaminergic terminals of the striatum was found in heroin addicts (Kish et al., 2001).

*In vivo* neuroimaging studies, which lack cellular resolution, showed reduced 6-FDOPA uptake in the dopaminergic terminals of the striatum of cocaine addicts during 10–30 days of abstinence (Wu et al., 1997). Interestingly, another marker of dopaminergic terminals in striatum, i.e., DAT levels, was found reduced in methamphetamine addicts (Chang et al., 2007) and in tobacco and marijuana smokers (Leroy et al., 2012). Extracellular DA release estimated using the 11C-raclopride displacement techniques indicated a lower DA tone in ventral striatum of cocaine (Martinez et al., 2009) and marijuana users (Volkow et al., 2014), the latter correlated with enhanced stress reactivity and irritability, confirming a hypodopaminergic state. Structural MRI showed a volumetric increase in the left nucleus accumbens in marijuana users (Gilman et al., 2014), enlarged striatum in methamphetamine users (Chang et al., 2007) and reduction in nucleus accumbens, anterior cingulated and orbitofrontal cortex in children exposed *in utero* to opioids (Walhovd et al., 2007), all findings in line with preclinical observations.

## CONCLUSION AND FUTURE RESEARCH

Addictive drugs induce structural plasticity in dopaminergic neurons. While a complete picture of structural changes associated to the different stages of the addiction cycle and its translational value in human is still lacking, differences among the main addictive drugs in producing either hypotrophic or hypertrophic response stand out, driven by the respective down or up regulations of BDNF and extracellular dopamine levels. These effects can be more conspicuous during neural development, as shown in offspring following *in utero* exposure or *in vitro* using embryo-derived cell cultures. Overall, structural changes appear to be related to some differences in targeting of reward and stress circuits that work in parallel to control motivation (Koob, 2013; Ikemoto and Bonci, 2014). These long term structural changes can be seen as substrates of “memory” traces (Nestler, 2013) that would eventually constitute a liability for drug taking relapse.

## Conflict of Interest Statement

The authors declare that the research was conducted in the absence of any commercial or financial relationships that could be construed as a potential conflict of interest.
